# Lapatinib Activates the Kelch-Like ECH-Associated Protein 1-Nuclear Factor Erythroid 2-Related Factor 2 Pathway in HepG2 Cells

**DOI:** 10.3389/fphar.2020.00944

**Published:** 2020-06-30

**Authors:** Noëmi Johanna Roos, Diell Aliu, Jamal Bouitbir, Stephan Krähenbühl

**Affiliations:** ^1^ Division of Clinical Pharmacology & Toxicology, University Hospital, Basel, Switzerland; ^2^ Department of Biomedicine, University of Basel, Basel, Switzerland; ^3^ Swiss Centre for Applied Human Toxicology (SCAHT), Basel, Switzerland

**Keywords:** lapatinib, HepG2 cells, mitochondria, reactive oxygen species, Nrf2, Keap1, glutathione

## Abstract

The receptor tyrosine kinase inhibitor lapatinib, indicated to treat patients with HER2-positive breast cancer in combination with capecitabine, can cause severe hepatotoxicity. Lapatinib is further associated with mitochondrial toxicity and accumulation of reactive oxygen species. The effect of lapatinib on the Kelch-like ECH-associated protein 1 (Keap1)-nuclear factor erythroid 2-related factor 2 (Nrf2) pathway, the major cellular defense pathway against oxidative stress, has so far not been studied in detail. In the present study, we show that lapatinib (2–20 µM) activates the Keap1-Nrf2 pathway in HepG2 cells, a hepatocellular carcinoma-derived cell line, in a concentration-dependent manner upon 24 h of treatment. Lapatinib stabilized the transcription factor Nrf2 at concentrations ≥5 µM and caused its nuclear translocation. Well-established Nrf2 regulated genes (*Nqo1*, *Gsta1*, *Gclc*, and *Gclm*) were upregulated at lapatinib concentrations ≥10 µM. Furthermore, cellular and mitochondrial glutathione (GSH) levels increased starting at 10 µM lapatinib. As a marker of oxidative stress, cellular GSSG significantly increased at 10 and 20 µM lapatinib. Furthermore, the gene expression of mitochondrial *Glrx2* and *SOD2* were increased upon lapatinib treatment, which was also observed for the mitochondrial SOD2 protein content. In conclusion, lapatinib treatment for 24 h activated the Keap1-Nrf2 pathway in HepG2 cells starting at 10 μM, which is a clinically relevant concentration. As a consequence, treatment with lapatinib increased the mRNA and protein expression of antioxidative and other cytoprotective genes and induced GSH synthesis, but these measures could not completely block the oxidative stress associated with lapatinib.

## Introduction

Receptor tyrosine kinases (TK) are transmembrane proteins that regulate important cellular pathways such as differentiation, proliferation, and apoptosis by phosphorylation of tyrosine residues (Yarden and Sliwkowski, 2001; Krause and Van Etten, 2005). Constantly activated or overexpressed TK can cause uncontrolled cell growth and are found in many tumor cells. Inhibitors of receptor tyrosine kinases (TKI), a class of small-molecule drugs, are efficient targeted anticancer drugs with a higher clinical benefit than non-targeted chemotherapeutic drugs (Amir et al., 2011).

Lapatinib is a TKI that is used in combination with capecitabine to treat patients with advanced or metastatic HER2-positive breast cancer (Moy et al., 2007). The TK HER2 (human epidermal growth factor receptor 2, also known as ErbB2) is overexpressed in up to 25% of invasive or metastatic breast cancers, and is associated with an unfavorable prognosis (Slamon et al., 1987). Lapatinib inhibits the intracellular TK domain of both HER1 (also known as epidermal growth factor receptor [EGFR] or ErbB1) and HER2. The most common adverse reactions of lapatinib therapy are diarrhea, nausea, rash, palmar-plantar erythrodysesthesia, and fatigue (Chu et al., 2007; Ryan et al., 2008). Furthermore, acute liver injury has been reported, a rare, but serious and potentially fatal adverse drug reaction prompting a FDA black-box warning in 2008 (Gomez et al., 2008). Lapatinib-induced liver injuries range from asymptomatic transaminase elevation, which affects approximately half of the patients treated with lapatinib in combination with capecitabine, to fatal cases of liver failure (Peroukides et al., 2011; Baselga et al., 2012; Azim et al., 2013). One possible explanation for lapatinib’s hepatotoxicity is mitochondrial dysfunction, as we have shown in our previous study (Paech et al., 2017). We found that lapatinib impairs mitochondrial function in a hepatocellular carcinoma-derived cell line (HepG2), which was accompanied by accumulation of reactive oxygen species (ROS) and release of cytochrome c from mitochondria into the cytosol inducing apoptosis. Dysfunctional mitochondria can generate ROS, mainly in the form of superoxide radicals (O_2_^•−^) (Brand, 2010; Brand, 2016). Mitochondrial (SOD2) and cytosolic (SOD1) superoxide dismutase convert mitochondrial O_2_^•−^ to H_2_O_2_, which is further detoxified to H_2_O and O_2_ by several antioxidative enzymes including glutathione-dependent hydrogen peroxidase (Balaban et al., 2005). An effective antioxidative defense is essential, since excess ROS can modify DNA bases, induce lipid peroxidation, and oxidize cellular proteins (Ott et al., 2007).

The nuclear factor erythroid 2-related factor 2 (Nrf2), a basic-leucine zipper transcription factor, and its negative regulator Kelch-like ECH-associated protein 1 (Keap1) form an inducible pathway for the defense against oxidative stress (Itoh et al., 1999; Yamamoto et al., 2018). Stimulated by ROS and electrophiles, Nrf2 induces the transcription of genes important for antioxidative defense and for phase II (conjugation) reactions (Reisman et al., 2009). Under unstressed conditions, Nrf2 is captured in the cytosol by Keap1, a cysteine-rich protein. Keap1 associates with Cullin 3 and RING-box protein to form a ubiquitin E3 ligase complex, which continuously ubiquitinates newly synthesized Nrf2 (Kobayashi et al., 2004). Ubiquitinated Nrf2 is efficiently degraded by the 26S proteasome, leading to low cellular Nrf2 levels under unstressed conditions. Specific cysteine residues of Keap1 can be oxidized and alkylated by H_2_O_2_ and electrophiles, respectively, leading to Keap1 inactivation (Suzuki et al., 2019). As a result, Keap1-dependent ubiquitination of Nrf2 decreases, Nrf2 is stabilized and translocates into the nucleus. Once in the nucleus, Nrf2 binds to antioxidant response elements located in the upstream promoter region of target genes and induces their transcription. Nrf2 regulates the transcription of genes involved in detoxification reactions (NADPH quinone oxidoreductase 1 [*Nqo1*], glutathione S-transferases [*Gst*]), glutathione (GSH) biosynthesis (glutamate-cysteine ligase [*Gclc*, *Gclm*]), and ROS elimination such as *SOD1* and *SOD2* (Reisman et al., 2009).

In the present study, we investigated the effect of lapatinib on the Keap1-Nrf2 pathway and its downstream effects. Specifically, we evaluated whether lapatinib stabilizes Nrf2 in a ROS-dependent fashion, studied the nuclear translocation of Nrf2 and determined the mRNA and protein expression of Nrf2-regulated genes.

## Material and Methods

### Reagents

The cell culture medium and supplements were purchased from Thermo Fisher Scientific (Basel, Switzerland). Lapatinib (SRP01210) was obtained from Sequoia research products (Pangbourne, United Kingdom). The other chemicals were purchased from Sigma-Aldrich (Buchs, Switzerland), if not indicated otherwise. For the LC-MS/MS measurements, we used HPLC grade solvents from Merck (Darmstadt, Germany), while the internal standards and the reference substances were obtained from Toronto Research Chemicals (Ontario, Canada). For western blotting, we used antibodies from Abcam (Cambridge, United Kingdom) and Santa Cruz Biotechnology (Heidelberg, Germany). The qPCR primers were produced by Microsynth (Balgach, Switzerland).

### Cell Culture

We cultured the human hepatocellular carcinoma cell line HepG2 in Dulbecco’s modified eagle medium (1.0 g/L D-glucose, 4 mM L-glutamine, 1 mM sodium pyruvate) supplemented with 10% (v/v) inactivated fetal bovine serum, 10 mM HEPES buffer (pH 7.4), 2 mM GlutaMAX, 1% (v/v) MEM non-essential amino acids solution (100x), and 100 U/ml penicillin/streptomycin. The cells were kept at 37°C in a humidified 5% CO_2_ cell culture incubator and passaged using TrypLE express enzyme (Thermo Fisher Scientific, Basel, Switzerland). We used the trypan blue exclusion method and the EVE automatic cell counter (NanoEnTek, Seoul, Korea) to determine the number of cells and their viability.

### Treatment of HepG2 Cells With Lapatinib

We treated HepG2 cells with 2, 5, 10, and 20 µM lapatinib for 2, 6, or 24 h. The stock solutions (1,000X) were prepared in dimethyl sulfoxide (DMSO) and stored at −20°C. Accordingly, we used 0.1% (v/v) DMSO as a negative control treatment.

### Membrane Toxicity

We used the ToxiLight bioassay kit from Lonza (Basel, Switzerland) to assess the cell membrane integrity of lapatinib-treated HepG2 cells. The assay measures the release of the intracellular enzyme adenylate kinase (AK) into the surrounding cell culture medium upon loss of cell membrane integrity. Released AK converts ADP, present in the detection reagent, to ATP, which creates a luminescent signal in the presence of luciferase. Briefly, we seeded HepG2 cells in a 96-well plate (10,000 cells/well). After lapatinib-treatment, we transferred 20 µl of cell culture medium from the treated HepG2 cells to an opaque-walled 96-well plate. After adding the AK detection reagent (50 µl/well), we incubated the plate for 5 min at room temperature, and recorded the luminescence using a microplate reader (Infinite 200 PRO, Tecan Group, Männedorf, Switzerland). As a positive control, we treated the cells with 0.1% (v/v) Triton X to induce cell lysis. Data were normalized to the ctrl. The AK release increased ~11.0-fold upon Triton X treatment compared to the negative control.

### Cellular ATP Content

We used the CellTiter-Glo luminescent cell viability assay (Promega, Dübendorf, Switzerland) to measure the cellular ATP content in lapatinib-treated HepG2 cells. After induction of cell lysis, the luciferase present in the CellTiter-Glo reagent generates a luminescent signal proportional to the ATP content. After lapatinib-treatment, we added an equal volume of CellTiter-Glo reagent directly to the lapatinib-treated HepG2 cells seeded in a white-walled 96-well plate (10,000 cells/well). The plate was placed on an orbital shaker for 2 min (600 rpm), incubated for further 8 min at room temperature, and the luminescence was recorded using the microplate reader. Data were normalized to control incubations containing 0.1% (v/v) DMSO. We used 0.1% (v/v) Triton X as a positive control, which caused a complete loss of viable cells (~100% ATP depletion).

### Mitochondrial Membrane Potential

We used the JC-1 mitochondrial membrane potential assay kit (ab113850, Abcam, Cambridge, United Kingdom) to determine the mitochondrial membrane potential (Δψ_M_) in lapatinib-treated HepG2 cells. Attracted by a high Δψ_M_, the cationic dye JC-1 (tetraethylbenzimidazolylcarbocyanine iodide) accumulates within polarized mitochondria, where it forms red fluorescent aggregates at high concentrations. Upon Δψ_M_ dissipation, JC-1 stops accumulating within mitochondria, and the JC-1 molecules, present as monomers at low concentration, emit green fluorescence. We performed the assay according to the manufacturer’s protocol. Briefly, HepG2 cells were seeded in a black-walled 96-well plate (15,000 cells/well) and covered with 100 µl of treatment solution. Thirty minutes before completed lapatinib-treatment, we added 2X JC-1 solution (40 µM) to each well (100 µl/well) and incubated for 30 min at 37°C protected from light. The cells were washed twice with pre-warmed 1X dilution buffer (100 µl/well). The last wash was left on the cells, and the fluorescence of both aggregates (λ_excitation_ = 475 nm, λ_emission_ = 590 nm) and monomers (λ_excitation_ = 475 nm, λ_emission_ = 530 nm) was measured using the microplate reader. We formed the ratio between the aggregate and monomer fluorescence (JC-1 ratio), which decreases upon Δψ_M_ depolarization, and calculated the fold-change relative to the negative control. Both JC-1 staining and final measurement were performed in the presence of lapatinib. Therefore, we spiked both staining solution and dilution buffer with the corresponding concentration of lapatinib. As a positive control, we treated the cells for 2 h with the ionophore uncoupler FCCP (carbonyl cyanide 4-(trifluoromethoxy) phenylhydrazone, 50 µM), which decreased the JC-1 ratio to ~38% of the control (DMSO 0.1%) levels.

### Mitochondrial O_2_^•−^ Accumulation

We used MitoSOX red (Thermo Fisher Scientific, Basel, Switzerland) to determine the accumulation of mitochondrial O_2_^•−^ in lapatinib-treated HepG2 cells. MitoSOX red accumulates in mitochondria and is selectively oxidized by mitochondrial O_2_^•−^ to a highly fluorescent oxidation product. We incubated lapatinib-treated HepG2 cells seeded in a black-walled 96-well plate (25,000 cells/well) with MitoSOX red (2.5 μM in PBS) for 10 min at 37°C under light protection. We recorded the fluorescence (λ_excitation_ = 510 nm, λ_emission_ = 580 nm) using the microplate reader, and subsequently lysed the cells with radio-immunoprecipitation assay (RIPA) buffer. The protein concentration was determined using a bicinchoninic acid assay with serum albumin as protein standard (Pierce BCA Protein Assay Kit, Thermo Fisher Scientific, Waltham, USA). Finally, we normalized the measured fluorescence to the protein concentration. As a positive control, we exposed HepG2 cells to amiodarone (25 µM for 24 h and 50 µM for 2 h or 6 h of exposure), a O_2_^•−^-generating mitochondrial toxicant (Felser et al., 2013). Amiodarone caused a ~1.5–2.4-fold increase of mitochondrial O_2_^•−^ accumulation.

### Cellular H_2_O_2_ Accumulation

We used the ROS-Glo H_2_O_2_ assay from Promega (Dübendorf, Switzerland) to measure the accumulation of H_2_O_2_ in lapatinib-treated HepG2 cells. The cellular H_2_O_2_ reacts directly with the H_2_O_2_ substrate, a luciferin derivative present in the H_2_O_2_ substrate solution, generating a luciferin precursor. The precursor is converted to luciferin upon addition of the ROS-Glo detection solution. Subsequently, the luciferase present in the luciferin detection solution generates a luminescent signal proportional to cellular H_2_O_2_ levels. HepG2 cells were seeded in a transparent 96-well plate (10,000 cells/well) and covered with treatment solution (80 µl/well). Six hours before the lapatinib-treatment was completed, we added H_2_O_2_ substrate solution to each well (20 µl/well), and incubated the plate for 6 h at 37°C. When the treatment with lapatinib was shorter than 6 h, the H_2_O_2_ substrate solution was added together with the treatment. After the incubation, we transferred 50 µl of supernatant from each well to a new white-walled plate and added ROS-Glo detection solution (50 µl/well). After incubation for 20 min at room temperature, we recorded the luminescence using the microplate reader. After the measurement, we lysed the cells with RIPA buffer and determined the protein concentration. Finally, the luminescent signal was normalized to the protein concentration. As a positive control, we treated the cells with menadione (50 µM), a H_2_O_2_-generating redox cycler (Deferme et al., 2013). The cellular H_2_O_2_ increased ~13.0–30.0-fold upon menadione treatment.

### Western Blotting

We assessed the expression of cellular proteins in lapatinib-treated HepG2 cells by western blotting. Briefly, we prepared protein lysates from lapatinib-treated HepG2 cells seeded in a six-well plate (2 x 10^6^ cells/well). RIPA buffer was used as lysis buffer. We determined the protein concentration of each sample, and prepared denatured and reduced protein samples by adding sodium dodecyl sulfate (SDS) and by boiling the samples at 95°C for 5 min. To separate the proteins according to their molecular weight, we loaded the protein samples (20 µg protein) on a NuPage 4–12% Bis-Tris Gel (Thermo Fisher Scientific, Basel, Switzerland), and ran a one-dimensional gel electrophoresis. NuPAGE LDS sample buffer was used as a loading buffer and PageRuler prestained protein ladder as a molecular weight marker, both purchased from Thermo Fisher Scientific (Basel, Switzerland). We used an eBlot L1 wet protein transfer system (Genescript, Piscataway, USA) and the eBlot L1 transfer sandwich (Genescript, Piscataway, USA) to transfer the separated proteins on a 0.2 µm nitrocellulose membrane (Bio-Rad, Cressier, Switzerland). To prevent non-specific background binding of the antibodies, we blocked the membrane for 1 h with 5% (w/v) non-fat milk in PBS containing 0.1% (v/v) Tween 20 (PBST) under constant shaking. The membrane was incubated overnight with primary antibody diluted in 5% (w/v) non-fat milk in PBST at 4°C. The following primary antibodies and dilutions were used: Nrf2 (1:10,000, ab62352), Keap1 (1:2,000, ab119403), SOD1 (1:5,000, ab51254), and SOD2 (1:5,000, ab74231). Antibodies against glyceraldehyde-3-phosphate dehydrogenase (GAPDH; 1:1,000, sc-365062) and Lamin B1 (1:1,000, ab229025) were used as loading controls.

After washing and incubating for 1 h with the corresponding horseradish peroxidase-conjugated secondary antibody (m-IgGK BP-HRP, 1:2,000, sc-516102; mouse anti-rabbit IgG-HRP, 1:2,000, sc-2357), we detected the protein bands using an ECL detection kit (Clarity Western ECL Substrate; Bio-Rad Laboratories, Cressier, Switzerland) and Fusion pulse 6 (Witec AG, Switzerland) as imaging system. The protein bands were visualized and quantified using the Evolution Capture software (Witec AG, Switzerland).

### Cytoplasmic and Nuclear Protein Extraction

We used the nuclear and cytoplasmic extraction kit (NE-PER Nuclear and Cytoplasmic Extraction Reagents, Thermo Fisher Scientific, Basel, Switzerland) to obtain nuclear and cytoplasmic protein fractions from lapatinib-treated HepG2 cells seeded in a six-well plate (500,000 cells/well). The cells were harvested, washed, and incubated in ice-cold cytoplasmic extraction reagent I. The buffer volumes were chosen according to the pellet size according to the manufacturer’s protocol. After incubating 10 min on ice, we added ice-cold cytoplasmic extraction reagent II to each sample, vortexed for 5 s, and incubated for 1 min on ice. We subsequently centrifuged the sample at 16,000 g for 5 min and obtained the cytoplasmic protein fraction (supernatant). The remaining pellet was further incubated in ice-cold nuclear extraction reagent on ice for 40 min with vortexing every 10 min. After centrifugation at 16,000 g for 10 min, we obtained the nuclear protein fraction (supernatant). The fractions were stored at −80°C until western blot analysis.

### Real-Time PCR

We extracted and purified RNA from lapatinib-treated HepG2 cells seeded in a six-well plate (2 x 10^6^ cells/well) using QIAshredders and the RNeasy mini kit (Qiagen, Hombrechtikon, Switzerland). Both extraction and purification were performed according to the manufacturer’s instruction. We determined the purity and RNA concentration of each extract using the NanoDrop One (Thermo Fisher Scientific, Basel, Switzerland). Then, we synthetized complementary DNA from 1 µg RNA using the Omniscript system (Qiagen, Hombrechtikon, Switzerland). SYBR green (Roche Diagnostics, Rotkreuz, Switzerland) and specific forward and reverse primers (**Table 1**; Microsynth, Balgach, Switzerland) were used to amplify the DNA templates.

**Table 1:** Specific forward and reverse primers used for real-time PCR.

Gene	Direction	Sequence (5′-3′)
*Nqo1*	Forward	5′-ATGGAAGAAACGCCTGGAGA-3′
	Reverse	5′-TGGTTGTCAGTTGGGATGGA-3′
*Gsta1*	Forward	5′-GAAGCCTCCCATGGATGAGA-3′
	Reverse	5′-AGCTTCACAACAGGCACAAT-3′
*Gclc*	Forward	5′-CATTGATTGTCGCTGGGGAG-3′
	Reverse	5′-CTGGGCCAGGAGATGATCAA-3′′
*Gclm*	Forward	5′-TGTATCAGTGGGCACAGGTA-3′
	Reverse	5′-GTGCGCTTGAATGTCAGGAA-3′
*Glrx1*	Forward	5′-CAGCCACCAACCACACTAAC-3′
	Reverse	5′-TGGTTACTGCAGAGCTCCAA-3′
*Glrx2*	Forward	5′-TCTGGGATGGAGAGCAATACA-3′
	Reverse	5′-TTCAAGCAGGTCCAGTTCCA-3′
*SOD1*	Forward	5′-TGTTGGAGACTTGGGCAATG-3′
	Reverse	5′-CAATGATGCAATGGTCTCCTGA-3′
*SOD2*	Forward	5′-TTTAGTCCCTGGTGTTCCCC-3′
	Reverse	5′-CTTCACCGAAAACTCCAGGC-3′
*Nrf2*	Forward	5′-TGAGCCCAGTATCAGCAACA-3′
	Reverse	5′-AGTGAAATGCCGGAGTCAGA-3′
*GAPDH*	Forward	5′-AGGTCGGAGTCAACGGATTT-3′
	Reverse	5′-TGACAAGCTTCCCGTTCTCA-3′

The real-time PCR was performed with three independent replicates on an ABI PRISM 7700 sequence detector (PE Biosystems, Switzerland) using the ViiA7 software (Life Technologies, Switzerland). We applied the comparative C_t_ method (ΔΔC_t_) to determine relative gene expression levels after normalizing to the housekeeping gene (*GAPDH*).

### Mitochondrial Isolation From HepG2 Cells by Magnetic Separation

Mitochondria were isolated from HepG2 cells using the human mitochondria isolation kit from Miltenyi Biotec (Solothurn, Switzerland). The isolation, which is based on magnetic separation using microbeads, was performed according to the manufacturer's protocol (Hornig-Do et al., 2009). In brief, we harvested, washed, and lysed 40 x 10^6^ HepG2 cells, and homogenized them with a Dounce homogenizer applying 90 strokes. The mitochondria were magnetically labeled by incubating the cell homogenate for 1 h with superparamagnetic microbeads conjugated to anti-TOM22 (translocase of outer mitochondrial membrane) antibodies. After the incubation, we applied the cell homogenate stepwise on pre-rinsed LS columns (Miltenyi Biotec, Solothurn, Switzerland) placed in the magnetic field of a MACS separator (Miltenyi Biotec, Solothurn, Switzerland). Pre-separation filters (30 µm) were used to remove cell aggregates. The magnetic field retains the labeled mitochondria in the columns, while the rest of the cell homogenate runs through. After three washing steps, we removed the columns from the separator and placed them in new microcentrifuges tubes. We flushed out the mitochondria by gently pushing a plunger into the column loaded with separation buffer. The mitochondrial pellet was obtained after centrifugation at 13,000 g for 2 min at 4°C using a microcentrifuge (Eppendorf Centrifuge 5415 R, Eppendorf, Schönenbuch, Switzerland).

### Cellular and Mitochondrial GSH and GSSG Content

We analyzed GSH and GSSG by liquid chromatography tandem mass spectrometry (LC-MS/MS) in lapatinib-treated HepG2 cells. GSH was also measured in mitochondria isolated from lapatinib-exposed HepG2 cells, while mitochondrial GSSG could not be analyzed due to stability problems during the measurement. As positive control, we treated HepG2 cells with 100 µM L-buthionine sulfoximine (BSO), which inhibits the GSH biosynthesis (Griffith and Meister, 1985). Upon 24 h-treatment, BSO reduced the cellular and mitochondrial GSH to ~9.0% and ~43.0% of the control levels, respectively. GSH ammonium salt-d_5_ (~10 mM, in H_2_O) and GSSG-^13^C_4_,^15^N_2_ (1 mM, in H_2_O) were used as internal standards. To prevent auto-oxidation of GSH and GSH ammonium salt-d_5_ during the sample preparation, we alkylated the thiol group with N-ethylmaleimide (NEM) forming GS-NEM and GS-NEM-d_5_ (Giustarini et al., 2013).

Briefly, 1 x 10^6^ lapatinib-treated HepG2 cells seeded in a six-well plate (1 x 10^6^/well) were harvested, washed, and immediately incubated in 250 µl alkylating solution (50 mM NEM in PBS) for 30 min on ice. During the washing step, we took an aliquot to determine the protein concentration of our samples. The same was done with the isolated mitochondrial pellet. Approximately 0.4 mg mitochondria were afterwards incubated in 100 µl alkylating solution. Both mitochondrial and cellular samples were extracted in a ratio of 1:4 (v/v) with internal standard solution, which consisted of 500 nM GSSG-^13^C_4_,^15^N_2_ and 500 nM GS-NEM-d_5_ in methanol. We kept the extracts at −20°C for 30 min to ensure protein precipitation. After centrifugation at 3,500 g for 10 min at 4°C, we transferred 250 µl of supernatant into a LC-MS/MS tube. Calibration lines of GS-NEM (250–0.25 µM) and GSSG (25–0.025 µM) were prepared in alkylating solution and extracted as described above.

An aliquot of 10 µl was injected into the LC-MS/MS system consisting of a Shimadzu HPLC (Kyoto, Japan) coupled to an API 4000 QTrap tandem mass spectrometer (ABSciex, Concord, Canada). The system was operated with the Analyst 1.6.2 software (AB Sciex, Concord, Canada). GS-NEM and GSSG were separated on a Symmetry C18 column (3.5 µm 100 Å, 4.6 mm x 75 mm; Waters, Eschborn, Germany) at a flow rate of 0.7 ml/min and a temperature of 45°C. We used H_2_O (phase A) and acetonitrile (phase B) both supplemented with 0.1% (v/v) propionic acid as mobile phases. We loaded the samples onto the analytical column using 7% mobile phase B and diluted it inline *via* a t-union with mobile phase A during the first 0.5 min of each run. Within the next 1.5 min, we linearly increased the gradient to 95% mobile phase B. We flushed the column with 95% mobile phase B for 1.5 min, and finally reconditioned the system with 7% mobile phase B for 1 min. The retention times of GSSG and GS-NEM were 2.00 min and 2.21 min, respectively. The analytes were positively charged by electro spray ionization and analyzed using scheduled multiple reaction monitoring. We applied an ion spray voltage of 5,500 V, and set the probe temperature at 700°C. The following mass transitions were used: 433.1 → 304.0 m/z for GS-NEM, 438.1 → 304.0 m/z for GS-NEM-d_5_, 613.1 → 355.0, 484.0, 231.1 m/z for GSSG, and 619.1 → 361.0, 490.0, 231.0 for GSSG-^13^C_4_^15^N_2_. Finally, the amount of GS-NEM and GSSG was normalized to the protein content of each sample.

### Statistical Analysis

The data are presented as the mean ± standard error of the mean (SEM) of at least three independent experiments. We performed one-way ANOVA statistical analysis with Dunnett's multiple comparison test using GraphPad prism version 8.2.1 (GraphPad Software, San Diego, USA). Significant values, indicated in the figures with an asterisk (*), were reached with a p-value <0.05 compared to control values (ctrl). We additionally performed two-way ANOVA statistical analysis followed by Sidak's multiple comparison test, whereby significant differences between groups (p-value <0.05) were indicated with hashtags (#).

## Results

### Membrane Toxicity, ATP Content, and Mitochondrial Membrane Potential in HepG2 Cells

**Figure 1:**
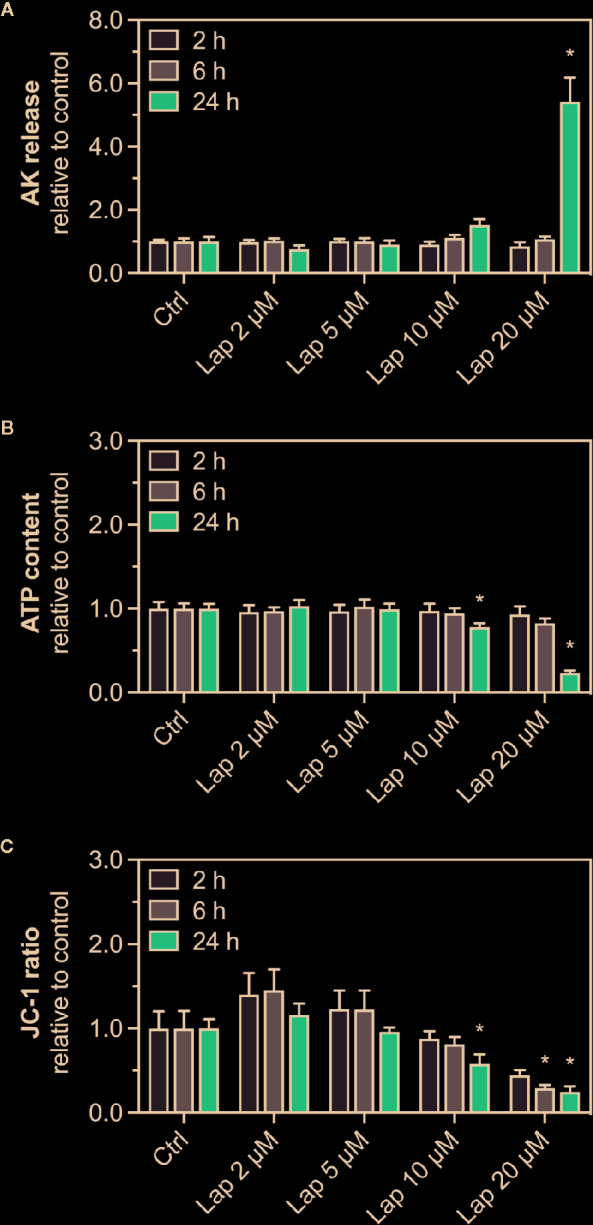
AK release, ATP content, and mitochondrial membrane potential in HepG2 cells. **(A)** Adenylate kinase (AK) release (cell membrane integrity marker), **(B)** cellular ATP content (cell viability marker), and **(C)** mitochondrial membrane potential (Δψ_M_) represented by the JC-1 ratio in HepG2 cells after treatment with 2–20 µM lapatinib (Lap) for 2, 6, and 24 h. The JC-1 ratio represents the ratio between JC-1 aggregates fluorescence (high Δψ_M_) to the fluorescence of JC-1 monomers (low Δψ_M_). Data are shown as fold increase relative to the negative control (0.1% DMSO, ctrl), and are the mean ± SEM of three independent replicates. *p < 0.05 versus negative control.

To confirm the findings of our previous study (Paech et al., 2017), we determined the release of AK, a marker for plasma membrane damage, into the surrounding cell culture medium from HepG2 cells treated with lapatinib for 2, 6, or 24 h (**Figure 1A**). The plasma membrane remained intact up to 6 h of lapatinib treatment. After 24 h of treatment, lapatinib started to damage the plasma membrane at 10 µM and caused a significant membrane damage at 20 µM. As a marker of cell viability, we measured the cellular ATP content in lapatinib-treated HepG2 cells (**Figure 1B**). While the cell viability was not diminished after 2 h of lapatinib treatment at any concentration, the cellular ATP level started to decrease after 6 h of treatment with 20 µM lapatinib (~17% ATP depletion). After 24 h, lapatinib significantly reduced the cell viability at 10 µM (~22% ATP depletion) and 20 µM (~76% ATP depletion).

ATP depletion could result from an impaired function of mitochondria, which could be reflected by a reduced Δψ_M_. We therefore measured the ΔψM (represented by the JC-1 ratio), a marker of mitochondrial function, in HepG2 cells treated with lapatinib for 2, 6, or 24 h (**Figure 1C**). The JC-1 ratio started to decrease at 10 µM lapatinib and was significantly reduced at this concentration after 24 h of treatment. A concentration of 20 µM lapatinib caused a significant JC-1 ratio reduction already after 6 h of exposure.

### Mitochondrial and Cellular ROS Accumulation

**Figure 2:**
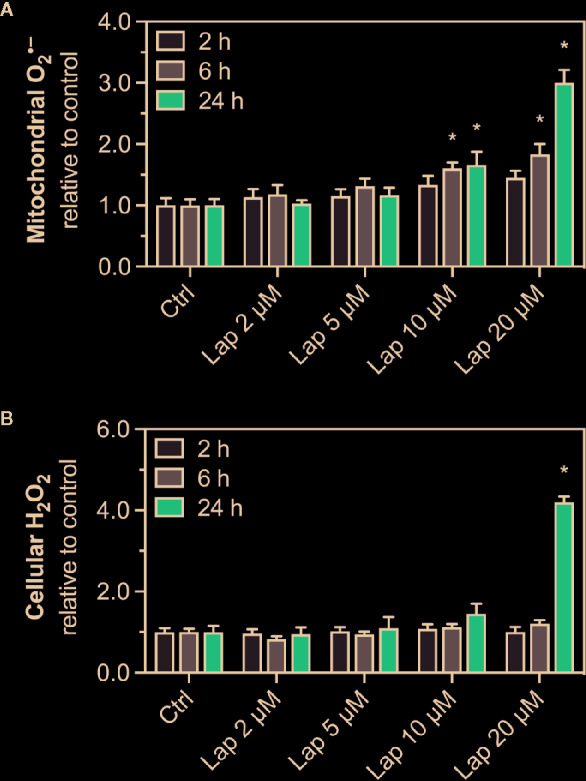
Accumulation of mitochondrial superoxide and cellular H_2_O_2_ in HepG2 cells. **(A)** Mitochondrial superoxide and **(B)** cellular H_2_O_2_ accumulation in HepG2 cells after treatment with 2–20 µM lapatinib (Lap) for 2, 6, and 24 h. Data are shown as fold increase relative to the negative control (0.1% DMSO, Ctrl), and are the mean ± SEM of at least three independent replicates. *p < 0.05 versus negative control.

The drop in the Δψ_M_ indicated mitochondrial dysfunction, possibly due to impaired function of the mitochondrial electron transport chain (Paech et al., 2017), which can be associated with increased generation of mitochondrial superoxide (O_2_^•−^) radicals (Brand, 2010; Brand, 2016). Mitochondrial O_2_^•–^ can spread into the cytosol once it is converted to H_2_O_2_. Thus, we measured the accumulation of mitochondrial O_2_^•^^–^ (**Figure 2A**) and cellular H_2_O_2_ (**Figure 2B**) in lapatinib-treated HepG2 cells. After 2 h of treatment, mitochondrial O_2_^•–^ accumulated at 10 and 20 µM without reaching significance. Upon lapatinib-treatment for 6 and 24 h, the mitochondrial O_2_^•–^ started to accumulate at 5 µM with a significant accumulation at both 10 and 20 µM. Cellular H_2_O_2_ started to accumulate upon 24 h-treatment at 10 µM, reaching significance at 20 µM. Overall, the effects were concentration- and time-dependent, and mitochondrial O_2_^•–^ started to accumulate at a lower lapatinib concentration than cellular H_2_O_2_.

### Activation of the Keap1-Nrf2 Pathway

**Figure 3:**
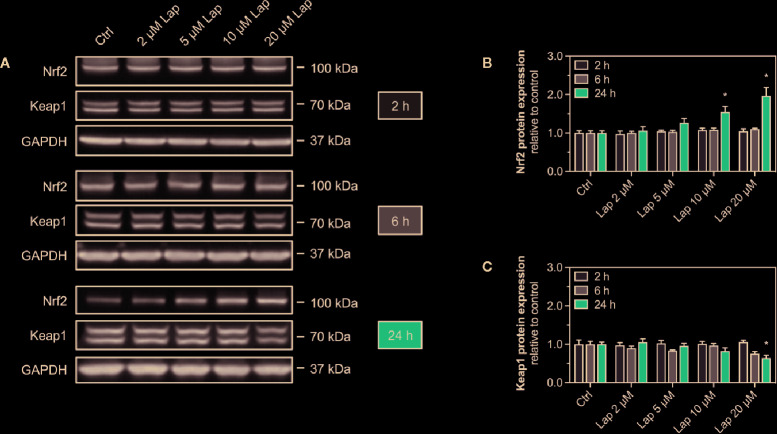
Protein expression of Nrf2 and Keap1 in HepG2 cells. **(A)** Representative western blot and quantification of **(B)** Nrf2 and **(C)** Keap1 protein expression in HepG2 cells after treatment with 2–20 µM lapatinib (Lap) for 2, 6, and 24 h. GAPDH represents the loading control. Data are shown as fold increase relative to the negative control (0.1% DMSO, Ctrl), and are the mean ± SEM of at least three independent replicates. *p < 0.05 versus negative control.

Accumulation of ROS can activate the Keap1-Nrf2 pathway, a major cytoprotective pathway against oxidative stress (Yamamoto et al., 2018). Concretely, H_2_O_2_ can induce disulfide bond formation between specific cysteine residues of Keap1 leading to Nrf2 stabilization (Suzuki et al., 2019). Thus, we assessed the cellular protein levels of Nrf2 in HepG2 cells treated with lapatinib for 2, 6, or 24 h (**Figures 3A, B**). No changes in the cellular protein levels were observed after 2 and 6 h of treatment. Nrf2 protein levels started to increase in HepG2 cells upon 24 h-treatment with 5 µM lapatinib and accumulated significantly upon treatment with 10 and 20 µM lapatinib (**Figures 3A, B**). Thus, lapatinib significantly increased the cellular protein levels of Nrf2 in a concentration-dependent manner, indicating Nrf2 stabilization. As shown in **Supplementary Figure 1**, lapatinib led to a numerical increase in Nrf2 mRNA expression, suggesting that increased Nrf2 gene transcription could contribute to the observed rise in the cellular Nrf2 protein content. In parallel, the cellular protein level of Keap1 (**Figures 3A, C**), which negatively regulates Nrf2, decreased in a concentration-dependent manner starting at 20 µM (after 6 h) and 5 µM lapatinib (after 24 h). Upon 24 h-treatment with 20 µM lapatinib, the Keap1 protein level was significantly reduced in HepG2 cells (**Figures 3A, C**).

**Figure 4:**
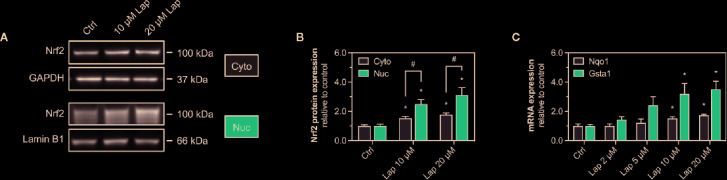
Activation of Keap1-Nrf2 pathway in HepG2 cells. **(A)** Representative western blots and **(B)** quantification of Nrf2 in nuclear (nuc) and cytoplasmic (cyto) fraction of HepG2 cells after 24 h-treatment with 10 and 20 µM lapatinib (Lap). Lamin B1 (nuclear fraction) and GAPDH (cytoplasmic fraction) represent the loading controls. **(C)** mRNA expression of the Nrf2-regulated genes *Nqo1* and *Gsta1* in HepG2 cells after treatment with 2–20 µM lapatinib (Lap) for 24 h. Data are shown as fold increase relative to the negative control (0.1% DMSO, Ctrl), and are the mean ± SEM of at least three independent replicates. ^*^p < 0.05 versus negative control, ^#^p < 0.05 versus the same concentration of lapatinib of the other experimental group.

After stabilization, the transcription factor Nrf2 translocates into the nucleus, where it binds to specific DNA promotors, the antioxidative response elements (Yamamoto et al., 2018). Therefore, we assessed the nuclear translocation of Nrf2 upon lapatinib-treatment (**Figures 4A, B**). We determined the protein levels of Nrf2 in the nuclear and cytoplasmic fraction extracted from HepG2 cells treated with 10 and 20 µM lapatinib (**Figure 4A**). Nrf2 protein levels significantly increased in both fractions in a concentration-dependent manner, whereby the accumulation in the nuclear fraction was significantly more pronounced (**Figure 4B**), indicating nuclear translocation of Nrf2.

### mRNA Expression of Nrf2-Regulated Genes

Once accumulated in the nucleus, Nrf2 induces the transcription of several genes involved in cellular antioxidative defense and cell protection mechanisms. We assessed the mRNA expression of *Nqo1* and *Gsta1*, two well-established Nrf2-regulated genes, in HepG2 cells upon lapatinib-treatment (**Figure 4C**). After 24 h of exposure, lapatinib induced the transcription of both genes in a concentration-dependent manner starting at 5 µM and reaching statistical significance at 10 and 20 µM for both genes. No such increase in mRNA expression of these genes was observed at 2 and 6 h of exposure to lapatinib (**Supplementary Figures 2A, B**).

### Involvement of ROS in the Activation of the Keap1-Nrf2 Pathway

**Figure 5:**
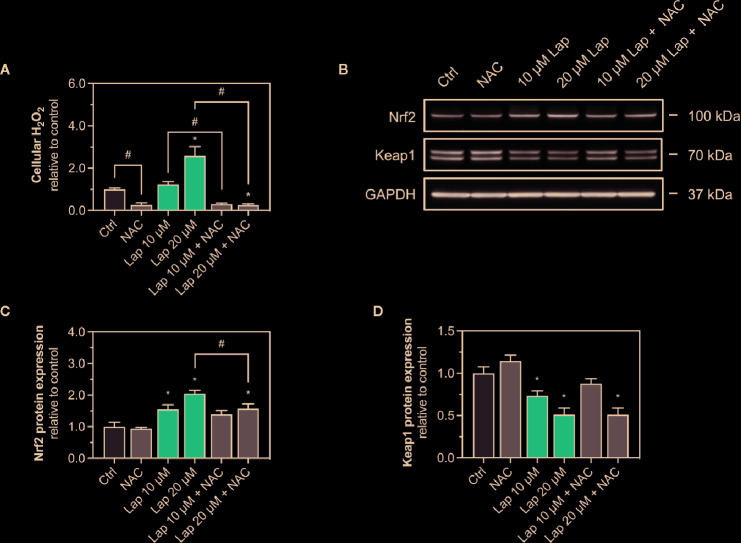
Involvement of ROS in the activation of the Keap1-Nrf2 pathway. **(A)** Cellular H_2_O_2_ accumulation in HepG2 cells after treatment with 10 and 20 µM lapatinib (Lap) with or without N-acetyl cysteine (NAC, 2 mM) for 24 h. **(B)** Representative western blot and quantification of **(C)** Nrf2 and **(D)** Keap1 protein expression in HepG2 cells after treatment with 10 and 20 µM lapatinib (Lap) with or without N-acetyl cysteine (NAC, 2 mM) for 24 h. Data are shown as fold increase relative to the negative control (0.1% DMSO, Ctrl), and are the mean ± SEM of at least three independent replicates. ^*^p < 0.05 versus negative control, ^#^p < 0.05 versus the same concentration of lapatinib of the other experimental group.

Since we observed accumulation of ROS upon lapatinib treatment, we assessed whether ROS accumulation is responsible for the activation of the Keap1-Nrf2 pathway induced by lapatinib. Thus, we used N-acetyl cysteine (NAC, 2 mM), a widely used antioxidant, to scavenge lapatinib-induced H_2_O_2_-accumulation after 24 h of treatment (**Figure 5A**). Co-treatment with NAC significantly reduced H_2_O_2_ accumulation at 10 and 20 µM lapatinib, qualifying NAC as an efficient ROS scavenger in our system. Co-treatment with NAC diminished the Nrf2 stabilization at the protein level at 10 and 20 µM after 24 h of treatment (**Figure 5B**). At 20 µM, the cellular Nrf2 content was significantly lower with a NAC co-treatment (**Figure 5C**). Nevertheless, the Nrf2 protein level increased at 10 and 20 µM lapatinib in the presence of NAC, suggesting that also activation mechanisms other than ROS accumulation are involved in the Nrf2 stabilization. The negative regulator Keap1 was less decreased in the presence of NAC at 10 µM lapatinib than in incubations without NAC (**Figures 5B, D**), suggesting that exposure to lapatinib could be associated with Keap1 oxidation.

### mRNA Expression of GSH-Related Genes and Levels of GSH and GSSG in HepG2 Cells and Isolated Mitochondria

**Figure 6:**
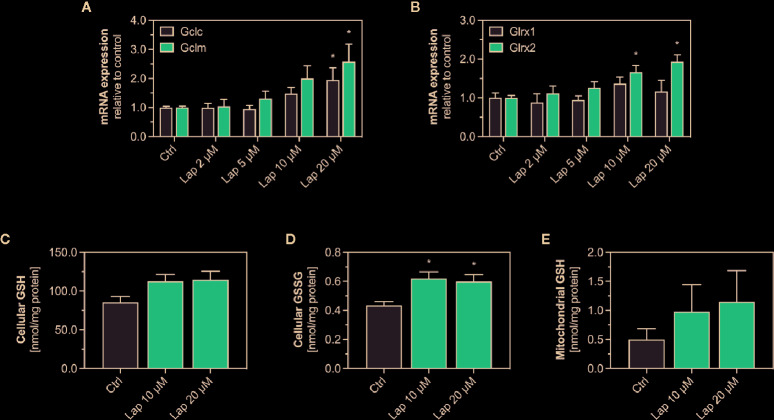
mRNA expression of GSH-related genes and levels of GSH and GSSG in HepG2 cells and isolated mitochondria. **(A)** mRNA expression of the Nrf2-regulated genes *Gclc* and *Gclm* as well as **(B)** *Glrx1* and *Glrx2* in HepG2 cells after treatment with 2–20 µM lapatinib (Lap) for 24 h. Data are shown as fold increase relative to the negative control (0.1% DMSO, Ctrl), and are the mean ± SEM of three independent replicates. *p < 0.05 versus negative control. Levels of **(C)** GSH and **(D)** GSSG in HepG2 cells after treatment with 2–20 µM lapatinib (Lap) for 24 h. **(E)** GSH levels in mitochondria isolated from HepG2 cells after treatment with 2–20 µM lapatinib (Lap) for 24. Data are the mean ± SEM of four independent experiments. *p < 0.05 versus negative control.

The product of the *Gsta1* gene detoxifies electrophiles through conjugation with glutathione (GSH), the most prevalent antioxidative molecule in hepatocytes (Griffith and Meister, 1985). The first step of the GSH synthesis is catalyzed by the glutamate-cysteine ligase (Gcl), which consists of a catalytic (Gclc) and a modifying subunit (Gclm). Since expression of both subunits is regulated by Nrf2, we measured the mRNA expression of *Gclc* and *Gclm* in lapatinib-treated HepG2 cells after 24 h (**Figure 6A**) and observed a concentration-dependent increase starting at 10 and 5 µM, respectively. The expression of both genes was significantly upregulated at 20 µM. We also measured the mRNA expression of glutaredoxin 1 (*Glrx1*) and 2 (*Glrx2*), which use GSH as a co-factor to reduce protein disulfides (**Figure 6B**). Interestingly, the transcription of the mainly cytosolic *Glrx1* was not significantly affected by lapatinib treatment at any concentration, while the expression of the mitochondrial *Glrx2* was significantly induced at 10 and 20 µM.

Lapatinb induced the transcription of *Gcl*, which encodes for the rate-limiting enzyme of GSH synthesis. GSH can directly detoxify ROS whereby being oxidized to GSSG. Thus, we measured GSH and its oxidized form GSSG in HepG2 cells treated with 10 and 20 µM lapatinib (**Figures 6C, D**). In HepG2 cells, we observed increased GSH levels at both concentrations (**Figure 6C**), and also significantly increased GSSG levels (**Figure 6D**). Since the GSH pool is compartmentalized within cells, we measured GSH specifically in mitochondria isolated from lapatinib-treated HepG2 cells (**Figure 6E**). Similar to the cellular GSH pool, also the mitochondrial GSH pool increased at both lapatinib concentrations investigated. The mitochondrial GSSG pool could not be analyzed due to stability issues during the measurement.

### mRNA and Protein Expression of Antioxidative Proteins

**Figure 7:**
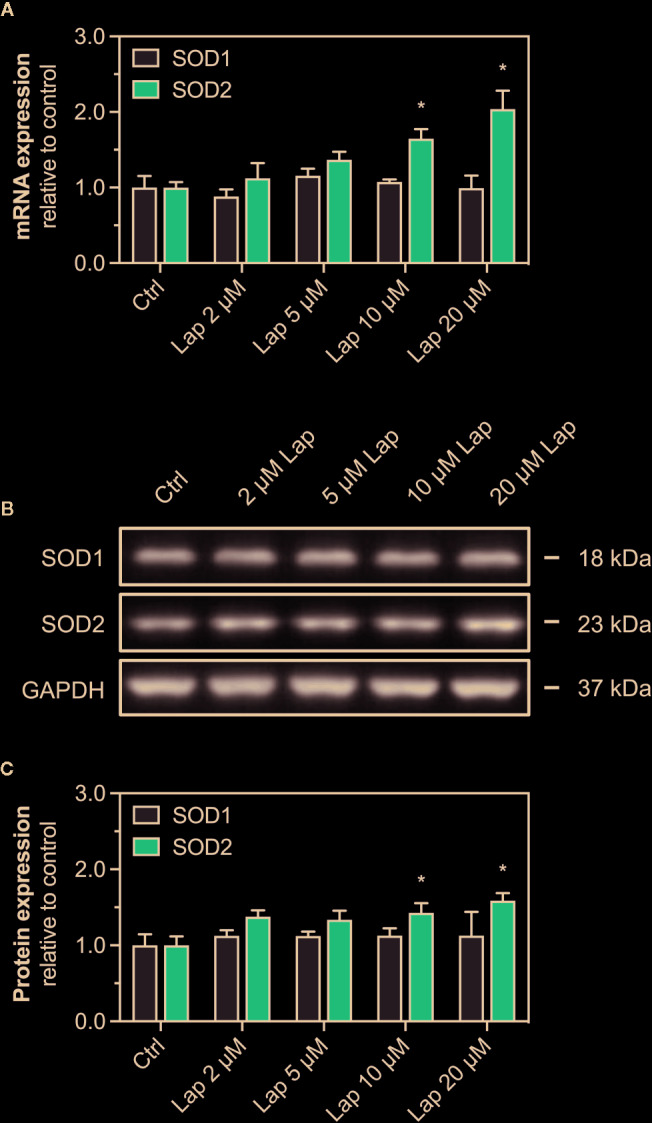
mRNA and protein expression of cytosolic SOD1 and mitochondrial SOD2. **(A)** mRNA expression of *SOD1* and *SOD2* in HepG2 cells after treatment with 2–20 µM lapatinib (Lap) for 24 h. **(B)** Representative western blot and **(C)** quantification of SOD1 and SOD2 in HepG2 cells after treatment with 2–20 µM lapatinib (Lap) for 24 h. GAPDH represents the loading control. Data are shown as fold increase relative to the negative control (0.1% DMSO, Ctrl), and are the mean ± SEM of three independent replicates. *p < 0.05 versus negative control.

Nrf2 also regulates the transcription of the antioxidative genes *SOD1* and *SOD2*, whose protein products are particularly important to detoxify mitochondrial O_2_^•−^ accumulated upon lapatinib-treatment. We measured the mRNA and protein expression of both superoxide dismutases in HepG2 cells after lapatinib-treatment for 2, 6, and 24 h. Regarding SOD1, neither mRNA (**Figure 7A** and **Supplementary Figure 3D**) nor protein expression (**Figures 7B, C** and **Supplementary Figure 3B**) were affected by lapatinib. In contrast, mRNA and protein expression of SOD2, an enzyme uniquely located in the mitochondrial matrix, significantly increased in a concentration-dependent manner at 10 and 20 µM lapatinib after 24 h of exposure (**Figures 7A–C**). In comparison, at 2 and 6 h of exposure to lapatinib, no such increase in SOD2 mRNA or protein expression was observed (**Supplementary Figures 3A, C, D**).

## Discussion

The present study provides evidence that the mitochondrial toxicant lapatinib activates the Keap1-Nrf2 signaling pathway in HepG2 cells. The nuclear translocation of Nrf2 induced the transcription of typically Nrf2-regulated genes including *Nqo1*, *Gsta1*, *Gclc*, and *Gclm*. Consequently, activation of the Nrf2 pathway resulted in increased cellular and mitochondrial GSH levels. Furthermore, lapatinib upregulated the gene expression of *Glrx2* and *SOD2*, which encode for mitochondrial antioxidative proteins. Activation of the Keap1-Nrf2 pathway, however, was not sufficient to avoid mitochondrial O_2_^•−^ and cellular H_2_O_2_ accumulation, GSSG formation, cell membrane damage, and ATP depletion upon 24 h-treatment with 10 and 20 µM lapatinib.

In our previous study, we showed that mitochondrial impairment is involved in the hepatotoxicity of lapatinib (Paech et al., 2017). In agreement with this earlier study, in the current study, we found a concentration-dependent membrane damage and reduced ATP pool in HepG2 cells after 24 h of treatment with lapatinib. We also observed that lapatinib dissipated the Δψ_M_ after 24 h of treatment, which was 24 h earlier than observed in our previous study. A decrease in Δψ_M_ could result from an inhibition of the mitochondrial electron transport chain and/or uncoupling of oxidative phosphorylation. In addition, we observed a concentration-dependent accumulation of mitochondrial O_2_^•−^ and accumulation of cellular H_2_O_2_ upon lapatinib-treatment, indicating that this drug causes oxidative stress. Taking into account that lapatinib is a mitochondrial toxicant, we can assume that ROS is generated first within mitochondria as O_2_^•−^, which is converted by SOD2 to H_2_O_2_. H_2_O_2_ can leave the mitochondria and accumulate in the cytoplasm, as observed in the current study. This sequence of events fits well with the findings in the current study, since we observed mitochondrial superoxide accumulation after 6 h and cellular H_2_O_2_ accumulation after 24 h of incubation with lapatinib. Inhibition of mainly complex I and III of the mitochondrial electron transport chain can stimulate the generation of mitochondrial O_2_^•−^ (Brand, 2010). However, in our previous study, lapatinib did not impair oxygen consumption by HepG2 cells, suggesting that lapatinib does not impair the mitochondrial electron transport chain (Paech et al., 2017). Importantly, mitochondria can produce ROS not only in the electron transport chain, but also at other sites such as for instance by the reaction of mitochondrial NADPH oxidase, monoaminoxidase, and/or α-glycerophosphate dehydrogenase (Zorov et al., 2014), with which lapatinib may interfere and induce mitochondrial ROS production.

Oxidative stress can inactivate Keap1 and increase its ubiquitination, resulting in decreased levels of Keap1, which is associated with decreased degradation and therefore increased cytoplasmic levels of Nrf2 (Zhang et al., 2005; Roos et al., 2020). Nrf2 stabilization, nuclear translocation, and upregulated transcriptional Nrf2 activity demonstrated the activation of this pathway by lapatinib in the current study. In addition, we assume that the observed rise in the cellular and mitochondrial GSH pools results from increased Nrf2 activity. Upregulation of GSH synthesis strengthens the cellular antioxidative capacity, which seems to be required since GSSG, the oxidized form of GSH, accumulated significantly in HepG2 cells.

In order to show the relationship between ROS accumulation and the Nrf2 activation, we co-treated HepG2 cells with lapatinib and the ROS scavenger N-acetyl cysteine (NAC). The co-exposure with NAC prevented lapatinib-associated ROS accumulation. Interestingly, in the presence of NAC, the Nrf2 accumulation was less pronounced when cells were exposed to lapatinib. This suggests that ROS accumulation upon lapatinib treatment can participate in the activation of the Keap1-Nrf2 pathway. The current study does not show, however, that activation of Nrf2 diminishes the toxicity of lapatinib. We have shown that in a recent publication for the mitochondrial toxicant benzbromarone, which was less toxic after stimulating the expression of Nrf2 by Keap1 knock-down (Roos et al., 2020).

Although ROS accumulation is a well-established mechanism of Keap1-Nrf2 pathway activation, we cannot exclude that lapatinib itself or reactive lapatinib metabolites contributed to the activation. Lapatinib is metabolized in the liver by cytochrome P450 enzymes, mainly by CYP3A4/5, to mostly pharmacologically inactive metabolites (Teng et al., 2010). HepG2 cells have only low levels of drug-metabolizing enzymes (Gerets et al., 2012; Berger et al., 2016). Thus, the formation of reactive metabolites in our cell system by cytochrome P450 enzymes is unlikely. Reactive metabolites could, however, be formed also upon oxidation by ROS. Oxidative cleavage of lapatinib's fluorobenzyl group (O-dealkylation) and/or oxidation of lapatinib's benzene rings could generate phenolic metabolites that could covalently bind to cysteine residues of Keap1 and subsequently stabilize Nrf2 (Teng et al., 2010; Castellino et al., 2012). This mechanism has been described for phenolic compounds that activate Nrf2 (Erlank et al., 2011). Furthermore, O-dealkylated lapatinib could be converted to a quinone imine reactive metabolite that forms GSH-adducts, demonstrating its ability to bind to cysteine residues (Hardy et al., 2014). This could, together with cellular accumulation of ROS, explain the need for increased GSH synthesis upon lapatinib treatment. Furthermore, covalent binding of reactive metabolites to cellular proteins can form haptens that could be phagocytosed by macrophages following hepatocyte breakdown and be presented on their surface by HLA proteins, which could activate T-cells. Indeed, HLA-DQA1*02:01 and DRB1*07:01 have been identified as risk factors for hepatotoxicity in patients treated with lapatinib (Spraggs et al., 2011; Spraggs et al., 2018). This mechanism cannot explain the observed hepatocellular toxicity in the current study, however, since no macrophages and T-cells were present in our assays.

Our results are in apparent contradiction to those reported by Eno et al. (2016). Eno et al. showed that 10 μM lapatinib for 24 h did neither impair oxygen consumption, nor activate the Keap1-Nrf2 pathway in HepG2 cells. Impairment of the cellular oxygen consumption and Nrf2 activation was only seen after transfection of HepG2 cells with CYP3A4, suggesting that a lapatinib metabolite was responsible for these findings. Indeed, O-dealkylated lapatinib reduced oxygen consumption and induced Nrf2 activation also in wild type HepG2 cells. In the current study, we started to see significant effects on mitochondrial function and Nrf2 activation at 10 μM lapatinib with more accentuated toxicity at 20 μM. Looking at the results on the NAD(P)H concentration in the publication of Eno et al. (Eno et al., 2016), it is clear that a numerical reduction without reaching statistical significance was already seen at 10 μM lapatinib. The difference in the findings of the two studies may therefore result mostly from the fact that we systematically studied lapatinib also at a concentration of 20 μM. Relevant CYP3A4 expression by the HepG2 cells can be excluded as shown in a previous publication by us (Berger et al., 2016).

In the current study, lapatinib started to activate the Nrf2 pathway at 5 µM with a significant effect at 10 and 20 µM, respectively. The maximal plasma concentrations after oral ingestion of a daily dose lapatinib (1,250 mg) combined with capecitabine are between 4.1–7.4 µM (Chu et al., 2007). Since lapatinib undergoes an extensive first-pass metabolism in the liver, the hepatic lapatinib concentrations are likely to be higher. Furthermore, the simultaneous intake of a fatty meal increased the bioavailability of lapatinib up to 4.3-fold (Koch et al., 2007; Koch et al., 2009). Also, the concomitant administration of 3A4-inhibitors such as ketoconazole increases the maximal plasma concentrations of lapatinib, a CYP3A4-substrate, up to 3.6-fold. (Medina and Goodin, 2008). Thus, we can conclude that the concentrations needed to activate the Keap1-Nrf2 pathway in our *in vitro* investigations can be reached in the liver of patients treated with this drug.

In conclusion, the mitochondrial toxicant lapatinib stabilized the transcription factor Nrf2 in HepG2 cells in a concentration-dependent manner, leading to the induction of important antioxidative and cell-protective Nrf2-regulated genes and upregulation of GSH synthesis. Beside the role of Nrf2 as a nuclear transcription factor, cellular Nrf2 accumulation can also be regarded as a marker of oxidative stress. The study illustrates that the hepatocellular toxicity of lapatinib is accompanied by activation of the Keap1-Nrf2 pathway. Beside the proposed immunological mechanism, mitochondrial toxicity with increased O_2_^•−^ production is an additional toxicological mechanism associated with liver injury of lapatinib.

## Data Availability Statement

All datasets for this study are included in the figshare repository: https://doi.org/10.6084/m9.figshare.12034608.v1.

## Author Contributions

NR and DA conducted the experiments, interpreted data, prepared figures and helped writing the manuscript. JB helped designing the study, supervised the lab work and helped in writing the manuscript. SK helped in designing the study, discussed and helped in the interpretation of the data and prepared the final version of the manuscript.

## Funding

The study was supported by a grant from the Swiss National Science Foundation to SK (SNF 31003A_156270).

## Conflict of Interest

The authors declare that the research was conducted in the absence of any commercial or financial relationships that could be construed as a potential conflict of interest.

## Supplementary Material

Supplementary Figure 1 mRNA expression of Nrf2 gene. **(A)** mRNA expression of the *Nrf2* gene in HepG2 cells after treatment with 2–20 µM lapatinib (Lap) for 24 h. Data are shown as fold increase relative to the negative control (0.1% DMSO, Ctrl), and are the mean ± SEM of three independent replicates. *p < 0.05 versus negative control.

Supplementary Figure 2 mRNA expression of Nqo1 and Gsta1 gene. mRNA expression of **(A)** Nqo1 and **(B)** Gsta1 in HepG2 cells after treatment with 2–20 µM lapatinib (Lap) for 2 and 6 h. Data are shown as fold increase relative to the negative control (0.1% DMSO, Ctrl), and are the mean ± SEM of three independent replicates. *p < 0.05 versus negative control.

Supplementary Figure 3 mRNA and protein expression of cytosolic SOD1 and mitochondrial SOD2. **(A)** mRNA expression of SOD2 after treatment with 2–20 µM lapatinib (Lap) for 2 and 6 h. **(B)** Representative western blot and quantification of **(C)** SOD1 and **(D)** SOD2 in HepG2 cells after treatment with 2–20 µM lapatinib (Lap) for 2 and 6 h. GAPDH represents the loading control. Data are shown as fold increase relative to the negative control (0.1% DMSO, Ctrl), and are the mean ± SEM of three independent replicates. *p < 0.05 versus negative control.
