# Supplementary figures and images for: Lapatinib Activates the Kelch-Like ECH-Associated Protein 1-Nuclear Factor Erythroid 2-Related Factor 2 Pathway in HepG2 Cells
Source: Front Pharmacol. 2020 Jun 30;11:944. doi: 10.3389/fphar.2020.00944 (PMC7339965; doi:10.3389/fphar.2020.00944)

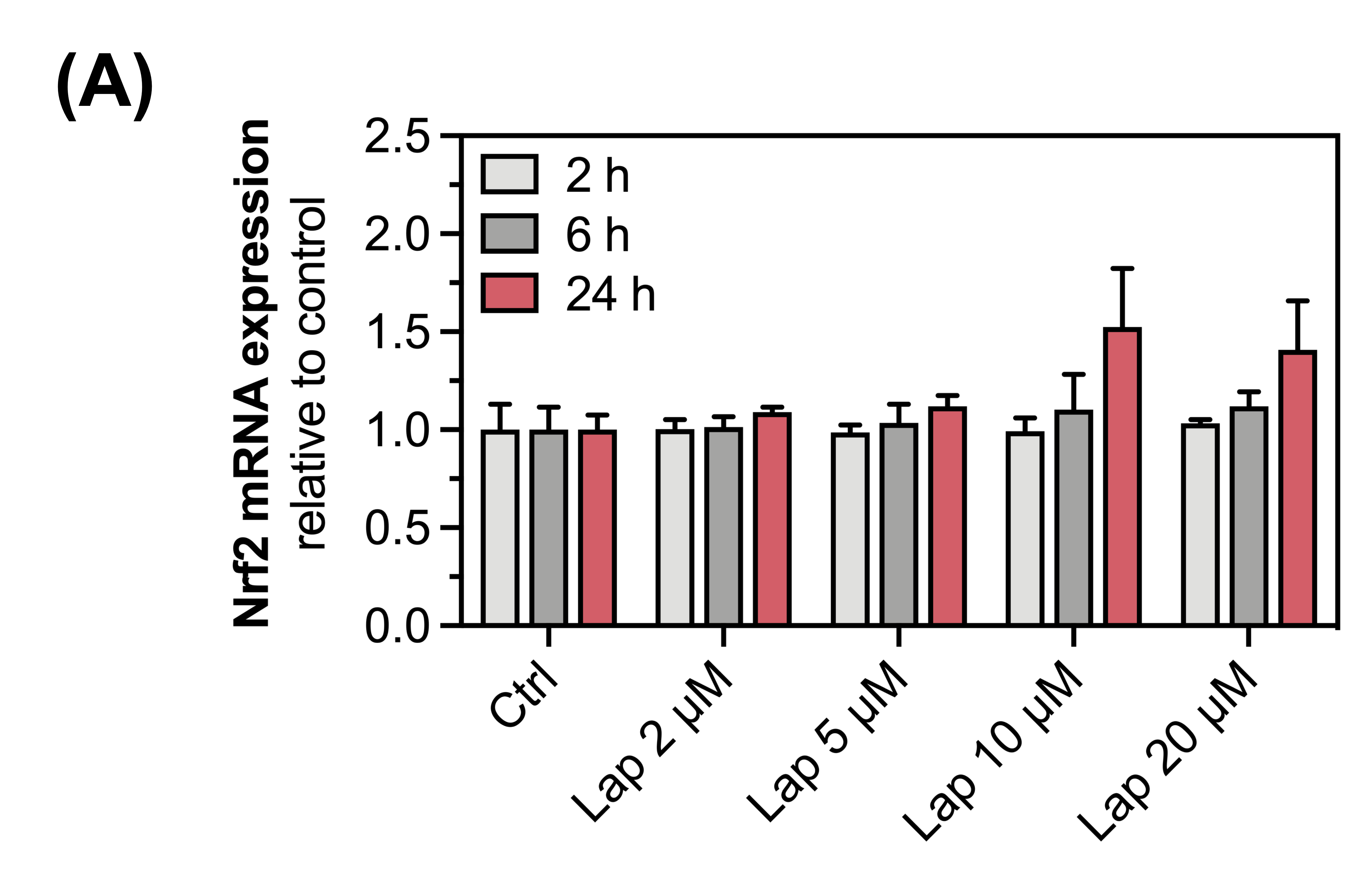

Supplement: Supplementary Figure 1 — mRNA expression of Nrf2 gene. (A) mRNA expression of the Nrf2 gene in HepG2 cells after treatment with 2–20 µM lapatinib (Lap) for 24 h. Data are shown as fold increase relative to the negative control (0.1% DMSO, Ctrl), and are the mean ± SEM of three independent replicates. *p < 0.05 versus negative control. [file Image_1.tiff]

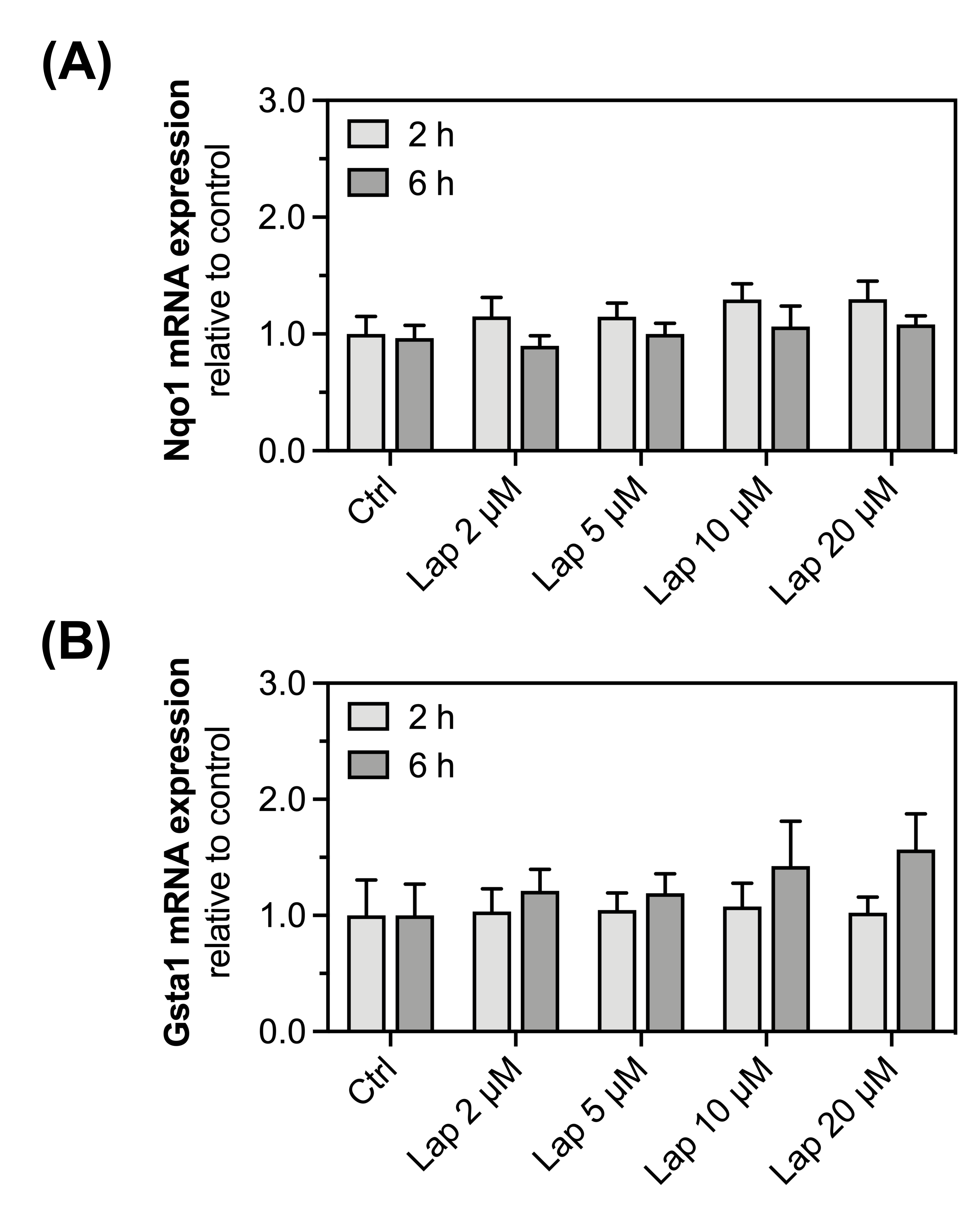

Supplement: Supplementary Figure 2 — mRNA expression of Nqo1 and Gsta1 gene. mRNA expression of (A) Nqo1 and (B) Gsta1 in HepG2 cells after treatment with 2–20 µM lapatinib (Lap) for 2 and 6 h. Data are shown as fold increase relative to the negative control (0.1% DMSO, Ctrl), and are the mean ± SEM of three independent replicates. *p < 0.05 versus negative control. [file Image_2.tiff]
